# Pooled Genome-Wide Analysis to Identify Novel Risk Loci for Pediatric Allergic Asthma

**DOI:** 10.1371/journal.pone.0016912

**Published:** 2011-02-16

**Authors:** Giampaolo Ricci, Annalisa Astolfi, Daniel Remondini, Francesca Cipriani, Serena Formica, Arianna Dondi, Andrea Pession

**Affiliations:** 1 Pediatric Unit, Department of Gynecologic, Obstetric and Pediatric Sciences, University of Bologna, Bologna, Italy; 2 Interdepartmental Centre for Cancer Research “G. Prodi,” University of Bologna, Bologna, Italy; 3 Department of Physics, University of Bologna, Bologna, Italy; 4 Interdepartmental Centre “L. Galvani”, University of Bologna, Bologna, Italy; Cincinnati Children's Hospital Medical Center, United States of America

## Abstract

**Background:**

Genome-wide association studies of pooled DNA samples were shown to be a valuable tool to identify candidate SNPs associated to a phenotype. No such study was up to now applied to childhood allergic asthma, even if the very high complexity of asthma genetics is an appropriate field to explore the potential of pooled GWAS approach.

**Methodology/Principal Findings:**

We performed a pooled GWAS and individual genotyping in 269 children with allergic respiratory diseases comparing allergic children with and without asthma. We used a modular approach to identify the most significant loci associated with asthma by combining silhouette statistics and physical distance method with cluster-adapted thresholding. We found 97% concordance between pooled GWAS and individual genotyping, with 36 out of 37 top-scoring SNPs significant at individual genotyping level. The most significant SNP is located inside the coding sequence of C5, an already identified asthma susceptibility gene, while the other loci regulate functions that are relevant to bronchial physiopathology, as immune- or inflammation-mediated mechanisms and airway smooth muscle contraction. Integration with gene expression data showed that almost half of the putative susceptibility genes are differentially expressed in experimental asthma mouse models.

**Conclusion/Significance:**

Combined silhouette statistics and cluster-adapted physical distance threshold analysis of pooled GWAS data is an efficient method to identify candidate SNP associated to asthma development in an allergic pediatric population.

## Introduction

Asthma is a chronic respiratory disease resulting from a complex interaction of multiple genetic and environmental factors. In the past decades, more than 200 asthma candidate genes have been identified using genetic association studies, positional cloning and knockout mouse approaches [1], but only in the recent years it has been possible to perform whole-genome investigations largely due to the genome-wide association studies (GWAS) [2]–[4], that have soon shown to be powerful tool to identify novel loci and susceptibility variants for common diseases.

Genome-wide association studies of pooled DNA samples were shown to be a valuable tool to identify in a fast, scalable and economical way candidate SNPs associated to a phenotype [5]–[7]. This method was applied to different SNP-array platforms and different diseases and QTL phenotypes, particularly those related to complex traits as intellectual and psychological abilities, multiple sclerosis or Alzheimer disease [8]–[13]. Many tools and analysis pipelines have also been developed to improve the ability to identify true associations among thousands of potential candidates [10], [14]–[16]. However, very few studies evaluated the efficacy of different methods in selecting candidate SNPs for second-stage validation, invariably reporting a high percentage of false positive results.

No study was up to now applied to allergic asthma, even if the very high complexity of asthma genetics is an appropriate field to explore the potential of pooled GWAS approach. This high complexity ensues from the frequent causal association of asthma with several other allergic phenotypes that may constitute a complex confounding factor for the identification of asthma susceptibility genes in the classical case-control design of GWAS (asthmatic *vs* healthy subjects).

To address both these issues, in this study we performed a pooled GWAS on pediatric allergic asthma, comparing asthmatic children to allergic subjects, and introducing a new method of analysis that incorporate silhouette statistics with quality control, physical distance and cluster-adapted thresholding, that reached 97% efficiency in selecting significant susceptibility loci associated to allergic asthma onset.

## Methods

### Pediatric patients cohort

A total of 269 children of white European descent, aged 6–18 years, with a diagnosis of allergic asthma and/or allergic rhinoconjunctivitis (RC) visited at outpatient clinic of Pediatric Allergologic Unit were included in the study. Patients were divided into two groups: patients with *asthma* (also including patients with both allergic asthma and rhinoconjunctivitis) and patients with *rhinoconjunctivitis* (RC), affected by allergic rhinitis or rhinoconjunctivitis who had never shown asthmatic symptoms. The mean age at the blood sampling was 10.6±3.6 yrs for *asthma* group and 9.8±3.7 for RC group (**Table 1**). Among patients included into *asthma* group, 95.33% developed asthmatic symptoms before 10 years of age and this evidence is confirmed by the most recent Italian and European studies which show that a percentage around 90% of subjects with full blown asthma become symptomatic before 10 years of age [17], [18]. Consequently we can assume that patients included in the RC group have a very low probability to develop asthma.

**Table 1:** Clinical, allergometric and spirometric characteristics of all the children included in the study.

	*Asthma*135 pts	*RC*134 pts	*p value*	*OR*
Age at blood sampling – mean ± SD (yrs)	10.6±3.6	9.8±3.7	NS	-
Onset age – mean ± SD (yrs)	5.2±2.8	6.2±3.1	NS	-
Sex (male/female)	2.65	1.44	0.02	1.84
Atopic Dermatitis (%)	24.4	31.3	NS	-
Other allergic manifestations (%)	15.6	14.2	NS	-
Total IgE – g. mean (kU/L)	423.6	245.9	0.0003	-
*Phleum p.* – sIgE g. mean (kU/L)	18.4	18.7	NS	-
*Phleum p. – s*IgE + (%)	74.1	69.4	NS	-
Grass pollen – SPT + (%)	72.6	70.9	NS	-
*D.pteronyssinus* - sIgE g. mean (kU/L)	6.6	3.1	0.02	-
*D. pteronyssinus* – sIgE + (%)	39.3	21.6	0.002	2.34
*D. pteronyssinus* – SPT + (%)	39.3	21.6	0.002	2.34
Baseline FVC %	101.2±16.0	104.0±11.4	NS	-
Baseline FEV_1_/FVC %	98.9±8.6	105.5±9.1	0.0005	-
Δ FEV_1_ %	5.6±5.2	2.3±4.5	0.002	-

Patients were divided into A*sthma* group and RC group, detected at enrolment. Allergic sensitization was reported for the main inhalant allergens: grass pollens (*Phleum p*.) and house dust mites (*D. pteronyssinus*) both as specific IgE and Skin Prick test. Main spirometric parameters were expressed as rate of predicted values (%). Δ FEV_1_ is the difference in FEV_1_ values before and after salbutamol administration.

An independent validation set, including 35 patients with asthma and 44 with rhinoconjunctivitis (RC), was selected in a second stage of the study. Clinical, allergometric and spirometric characteristics of the patients were comparable to the ones included in the first stage of the study.

### Ethics Statement

The study was conducted according to the principles expressed in the Declaration of Helsinki, and approved by Ethic Committee of University Hospital – S. Orsola-Malpighi of Bologna (*AllerGene* protocol no. 134/2008/U/Tess). Written informed consent was obtained from all the parents or guardians of the minors involved in the study.

### Allergometric assays

Patients' allergometric assessment was evaluated through the determination of specific IgE levels (UNICAP1000, Phadia, Uppsala, Sweden) and by performing Skin Prick tests (Lofarma, Milan, Italy). Skin prick tests were performed for the main inhalant allergens: Grass pollen, *Parietaria mix*, *Compositae mix*, birch pollen, hazelnut pollen, *D. pteronyssinus*, *D. farinae* and *Alternaria a*., *Cladosporium*, cat's epithelium and dog's dandruff. Referring to the ACAAI Practice Parameters [19] wheals with a diameter ≥ histamine (described as + + for a convention) was considered positive. For each patient total IgE and specific IgE for the main inhalant (*Cynodon d., Phleum p*., *Ambrosia e*., *Artemisia v*., *Parietaria J*., *Betula v.*, *Corylus a*., *Olea e*., *D. pteronyssinus*, *D. farinae*, *Alternaria a*., dog's dandruff, cat's epithelium) were determined; we considered positive specific IgE values higher than 1 kU/L to avoid immunological cross-reactivity. Only the sensitization for the most represented allergens has been reported (**Table 1**).

### Assessment of pulmonary status of patients

Pulmonary status of patients was evaluated by performing spirometric tests (ZAN100, nSpire Health GmbH, Germany). In asthmatic children lung function tests were performed both for diagnostic purposes and to establish the efficacy of pharmacological therapy (inhalatory corticosteroids), while in patients with RC to exclude a silent pulmonary inflammatory condition. Measurements of lung function were performed both for *asthmatic* and *non asthmatic* children and **s**pirometric parameters were evaluated as variation between values at baseline and after administration of rapid-acting bronchodilator (e.g. after 200–400 µg salbutamol), both to demonstrate the reversibility of lung function abnormalities and to exclude an eventual airflow limitation also in patient with normal value at baseline. The measured variables were evaluated according to the GINA guidelines [20] and were reported the most representative indicators of asthmatic condition: FVC (forced vital capacity), FEV_1_ (forced expiratory volume in one second), FEV1/FVC ratio (**Table 1**).

### Clinical statistical analysis

Statistical significance for age at blood sampling and onset age of symptoms was evaluated by performing Mann Whitney test. The Chi squared test was performed to calculate the significance of gender differences, prevalence of atopic dermatitis and other allergic manifestations and the prevalence of sensitization to the major inhalant allergens, for which Odds Ratio (OR) was also estimated. Total and specific IgE values, expressed as geometric mean, were evaluated by Fisher exact test. Values of *P*<0.05 were considered as significant. Spirometric measurements were reported as average values and their significance was evaluated by Mann Whitney test (**Table 1**).

### Power analysis

Sample dimension was calculated by modeling the LD coefficient (D') as a function of the genetic distance between the associated marker allele and the disease locus (assuming the worst-case scenario of the disease locus being located midway between two markers) and of the number of generations elapsed (G) since the mutation was introduced. G was chosen as representative of an outbred population (G = 500), and the recombination fraction between marker and disease locus was estimated from the number of markers on the array (roughly 500,000).

Sample dimension was then estimated, assuming a frequency of the predisposing allele of 0.15, a disease prevalence of 0.4 (prevalence of asthma in the allergic population is ≥40%) [21], a genotype relative risk of 1.7 (for both the homozygote and heterozygote), and a marker frequency of 0.2 (taken from Affymetrix product description). Under these conditions it is possible to identify such a susceptibility allele with a statistical power of 80%, a type I error rate of 0.05, and a study of 136 cases and 136 controls. The estimates were calculated using the Genetic Power Calculator web tool [22].

### DNA extraction and sample pooling

DNA was extracted from all the blood samples with Nucleospin DNA kit (Macherey & Nagel, Duren, Germany) and individual DNA concentration was determined in triplicate with the Quant-iT Pico-Green dsDNA Assay Kit (Invitrogen, Milan, Italy). These triplicate values were used to calculate a mean concentration for each sample. Samples were excluded from the analysis if the coefficient of variation between the three replicates was <0.1.

DNA samples were assigned to the *Asthma* group if displaying symptoms of asthma, alone or associated to other allergic phenotypes, including RC, and assigned to the *RC* group if displaying rhinitis or rhinoconjunctivits alone or associated to other allergic phenotypes, excluding asthma. Each of the two groups was subdiveded into 4 independent groups of samples, each containing 31-36 individuals. Individual DNA samples were then added to their respective pools in equivalent molar amounts. The design of multiple replicate pools of distinct individuals was chosen as it decreases quantification error by reducing quantification error variance proportionally to the number of times a single pool is independently constituted (3 times), and measurement error variance by the number of independent analysis (24 times) [23].

### Genome Wide SNP genotyping

Pooling was performed three times to account for pipetting variability. Each of these replicates was genotyped on Mapping 500K arrays (Affymetrix, Santa Clara; CA) following manufacturer's instructions. Detection rates were calculated with GTYPE 4.1 (Affymetrix) using the MPAM (*Modified Partitioning Around Medoids*) MDR algorithm, that represents the number of SNPs that passes the MPAM discrimination filter/total number of SNPs. MDR ± SD was 98.3±1.8. Raw data were deposited in the GEO repository with the GSE24481 reference number.

### Analysis of pooled SNP genotyping data

Probe intensity data was taken directly from the CEL file and used to calculate a Relative Allele Signal (RAS) score by the MPAM algorithm implemented in the SNP-MaP script (http://sgdp.iop.kcl.ac.uk/oleo/affy). This method calculates the average of RAS1 (sense) and RAS2 (antisense) values as a quantitative index of allele frequencies in pooled DNA [6].

Probes were filtered by overall average RAS (0.1<RAS<0.9) and AAD (*Average Absolute Difference*, AAD<0.28) to exclude data with low Minor Allele Frequency and high variability.

To control for possible confounding bias due to population stratification we applied Principal Component Analysis (PCA) on the RAS values from the Top 500 ancestry-informative SNPs identified by Drineas et al. [24], selected from a subset Population Reference Sample (POPRES) representative of 11 different populations and analyzed on Mapping 500K arrays (Affymetrix). PCA was done also in comparison to allele frequencies in CEU, YRI, CHB and JPT population taken from Hapmap data.

We used silhouette score [25], [26] to assess SNP significance. Silhouette score is a measure to quantify the goodness of data clustering, defined as the average silhouette calculated over all cluster elements. For the *i*-th cluster element, silhouette is defined as
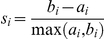
where *a_i_* is the average (Manhattan) distance of the i-th element from each element of the cluster, and *b_i_* is the average distance over the other clusters (in our case we have only two clusters, Asthma and RC). Silhouette values range from –1 (bad clustering) to 1 (optimal clustering).

We chose not to use an F test statistics because very probably the RAS values (ranging from 0 to1) and in particular the extreme values, do not fulfil the condition of gaussianity necessary for the application of the test. Also the absolute values of the differences were not considered since they do not take into account sample variability.

For the first step of our analysis we considered 1% top-scoring probes (about 3000 probes selected). We decided not to choose a too conservative threshold for single-probe significance (e.g. by comparison with silhouette values of label-reshuffled data) but we tried to evaluate a multi-probe significance by looking at the presence of significant SNP “blocks” (i.e. clustered together along the chromosome) that likely reflect the presence of LD blocks including a large number of (even weakly) significant probes. We evaluated the robustness of our results considering different starting datasets (5000 and 7000 top-scoring probes) and different parameters for clustering (chromosomal distance threshold from 20 Kb to 35 Kb, see below) obtaining a good agreement in the final SNP lists.

SNPs were mapped onto the Human Reference Sequence *hg19* GrCh37 assembly (UCSC Genome Browser). Then we clustered probes with genomic distance <30 Kb, and we considered only probes belonging to at least a 2-probe cluster. For cluster crossvalidation, we assigned randomly the calculated silhouettes to all probes, and then repeated all the analysis steps up to calculating average cluster silhouette for each cluster size (100 times): average cluster silhouette obtained by reshuffling was compared to the real average cluster silhouette values, producing the final list of significant SNPs.

### Validation of candidate SNPs

The LD structure of the significant clusters was visualized by GOLD heatmap and analyzed using Haploview software v. 4.1. on Caucasian phased genotypes from the International Hapmap Project (www.hapmap.org). SNAP tool (SNP annotation and proxy search) was used to find genes proxy to significant SNP clusters by Linkage Disequilibrium, by setting r^2^ threshold to 0.8 and distance to 500 Kb, on Hapmap 22 release data (http://www.broadinstitute.org/mpg/snap/). For each cluster average silhouette score was reported.

Genotyping of the Top scoring SNPs for the validation study was performed using the MassArray system on a Sequenom MALDI-TOF device (Sequenom Inc., San Diego, CA) on the entire dataset. Primer sequences and PCR conditions are available upon request. Call rate was 100% for all SNPs and samples analyzed. SNPs were assessed for Hardy-Weinberg equilibrium and analyzed by a two-sided Fisher exact test to compare allele and genotype frequencies between cases and controls. Strength of association was estimated by conditional OR ±95%CI. Quality controls and allelic and genotypic association tests were performed using the SNPator package (http://www.snpator.org). Genotyping of the 24 top scoring SNPs associated with known genes was also performed on the Sequenom system for the independent validation set.

### Gene expression

Gene expression analysis was performed on GSE6858 and GSE1301 Gene Expression Omnibus databases (http://www.ncbi.nlm.nih.gov/geo/), that report gene expression profiling data on two relevant mouse models of experimental asthma, analyzed on MG430 2.0 and MOE430A Affymetrix arrays, respectively. The first analyzes experimental asthma induced in BALB/c mice by sensitization and challenge with the allergen ovalbumin [27], while the second one explores the response of whole lungs of BALB/c mice to asthma induced by house dust mite sensitization and challenge, *vs* control mice. CEL files were downloaded, normalized with *rma* algorithm and filtered based on gene expression level (>5 in the log2 scale, at least 50% of samples) and InterQuartile Range (IQR >10% of average total IQR). Differential genes between allergen-challenged and control mice were selected by a modified *t*-test implemented in *limma* package [28], with *P*<0.05 cutoff. Probe sets corresponding to mouse orthologs of human genes were identified by the Affymetrix annotation in the NetAffx database.

## Results

We recruited 269 children of self-reported white European ancestry, aged 6 to 18 years with a well documented diagnosis of allergic asthma and/or *rhinoconjunctivitis* (RC). Clinical, phenotypic and spirometric data of the subjects recruited in the *AllerGene* study are summarized in **Table 1**. Among the patients of the two groups no differences in the prevalence of atopic dermatitis and other allergic manifestations could be highlighted, while male gender was associated with asthmatic phenotype (*P* = 0.02, OR 1.84). Asthmatic patients have higher total IgE values (*P = *0.0003), higher specific IgE values (*P* = 0.02) and higher prevalence of sensitization to house dust mite (*P* = 0.002) than patients with RC without asthmatic symptoms. According to pulmonary function tests at baseline, no differences in FVC were underlighted between *Asthma* and RC groups, while asthmatic patients showed a lower value of FEV_1_/FVC (*P* = 0.0005). Spirometric tests performed after administration of salbutamol revealed significant higher variation of FEV_1_ in patients with asthma (*P* = 0.002).

**Figure 1:**
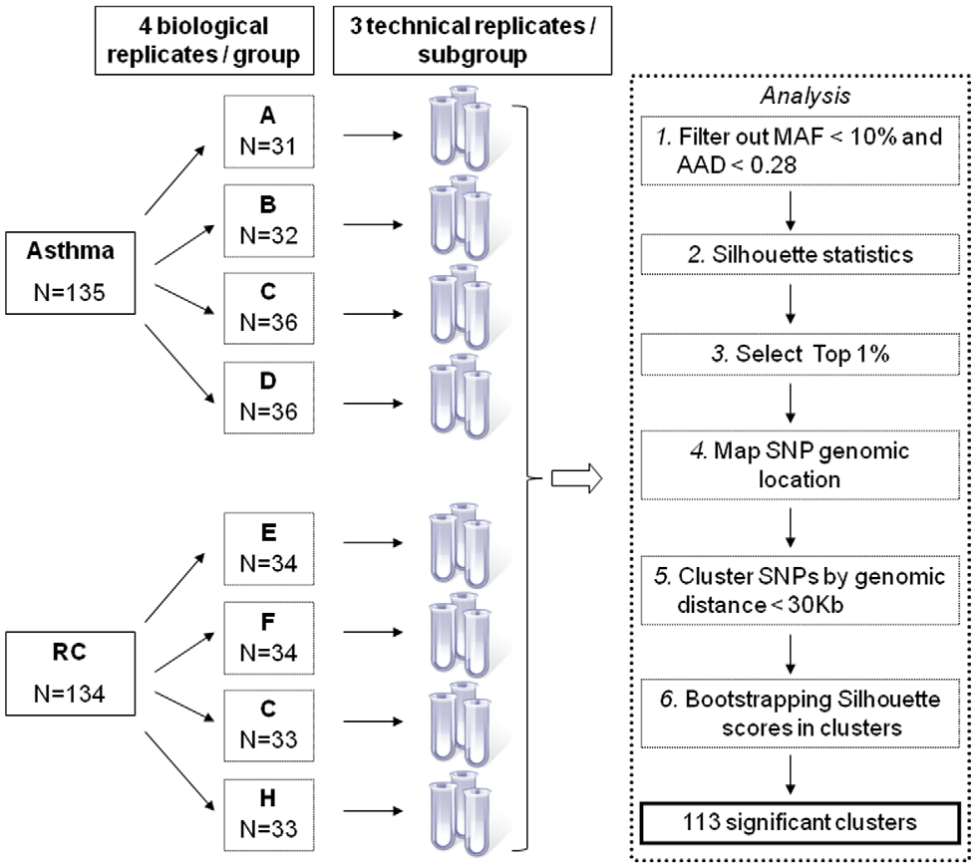
Data analysis pipeline. Asthma and RC samples were divided in 4 pools of 31–36 elements, labeled and hybridized to Mapping 500K SNP arrays. Different pools were replicated three times to account for tecnical and manual variability. Data were filtered, silhouette statistics was applied and top 1% SNP was mapped onto UCSC genome browser and only clusters of at least two SNPs with a genomic physical distance <30 Kb were retained. Cluster-adapted silhouette threshold was calculated by averaging reshuffled random cluster silhouette scores.

GWAS analysis by SNP arrays was undertaken on pooled DNA samples from 269 subjects of the study participants on the Mapping 500K Affymetrix platform. Patients were classified into *Asthma* (135) and *RC* (134) groups, and further subdivided into 4 subgroups considered biological replicates. Pooling was performed in triplicate, to account for technical variability (**Figure 1**). This multiple-pool strategy optimizes the power of the analysis by reducing the variance of each source of experimental error [23].

To control for possible stratification bias due to population structure we applied Principal Component Analysis to a list of 500 top scoring SNPs identified as ancestry-informative in a subset of the Population Reference Sample [24]. PCA showed that there is no recognizable substructure inside our data *(Asthma* and RC pools are mixed up), suggesting that no bias related to population structure was inflated into the data (**Figure S1 in File S1**). As expected the distribution of allele frequencies inside the pools is compatible with CEPH ancestry (Northern and Western european, **Figure S1 in File S1**).

Data reliability was assessed by checking Pearson correlation coefficient C among samples: technical replicates resulted more correlated among themselves (C>0.96). We also checked single probe average variance a) for triplicates, b) for classes (*Asthma*/RC) and c) for all arrays, confirming data homogeneity (µ_a_ = 0.21; µ_b_ = 0.23; µ_c_ = 0.25; σ_a_ = 0.54; σ_b_ = 0.62; σ_c_ = 0.58) (**Figure S2 in File S1**).

Probes were then filtered by overall average RAS (*Relative Allele Signal*) and AAD (*Average Absolute Difference*) to exclude data with low Minor Allele Frequency and excessive variability (78.6% selection) (**Figure S3 in File S1**).

**Figure 2:**
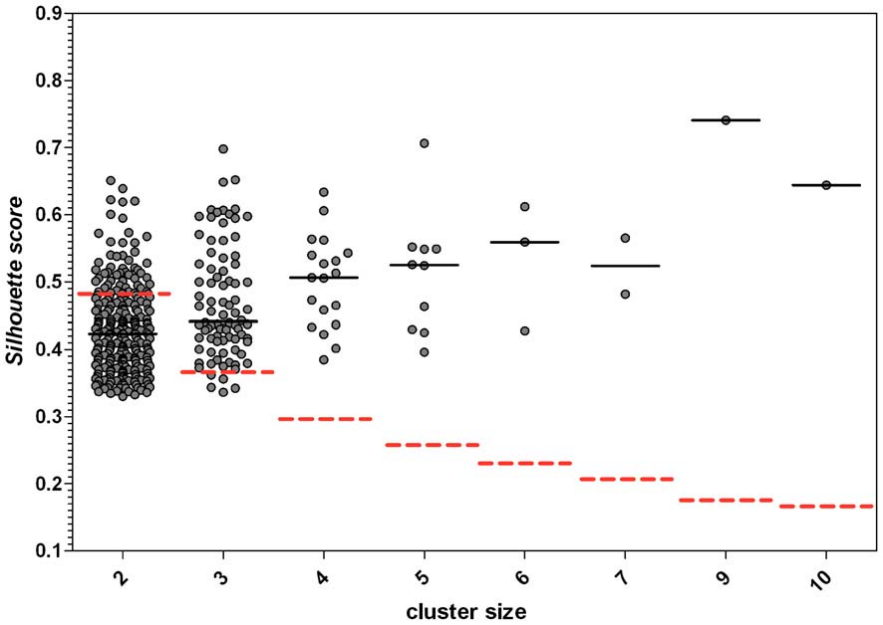
Details of cluster crossvalidation results. Silhouette average score for each cluster is shown as black circles and silhouette average score threshold calculated by random score reshuffling as red dashed lines.

RAS values were analyzed by a Silhouette score statistic, that empirically showed to perform well in pooled-GWAS studies [14], and mapped to the human Reference Sequence *hg19* assembly. Significant SNPs were selected as clusters of top 1% significant silhouette scores within a genomic distance of 30 Kb, and crossvalidated to control for type I errors by random reshuffling average silhouette scores. Only clusters showing an average silhouette score above the cluster-adapted threshold were called significant (**Figure 2**).

Of the 113 significant clusters identified, SNPs representative of the first 37 clusters selected as the top-scoring (Silhouette *P*<0.001) were validated by individual genotyping by the MassArray genotyping system (Sequenom). Individual allelic and genotypic frequencies showed significant association with asthma for 36 SNPs (allelic *P*<0.05. **Table S1–S2 in File S1**), showing a 97% concordance between pooled GWAS and individual genotyping results. More than half of the identified candidate genetic loci were in significant LD with known genes from the Refseq database, or directly located inside introns or coding sequences (**Table S1 in File S1**). The most significant cluster as determined by average silhouette score statistic is located inside the coding sequence of *Complement component 5* gene (*C5*) that is already known as an asthma susceptibility gene reported in previous studies [29]. It is localized on chromosome 9q33.2, in LD with two other genes that regulate inflammation and significantly associated with allergic asthma, *GSN* and *RAB14*.

**Figure 3:**
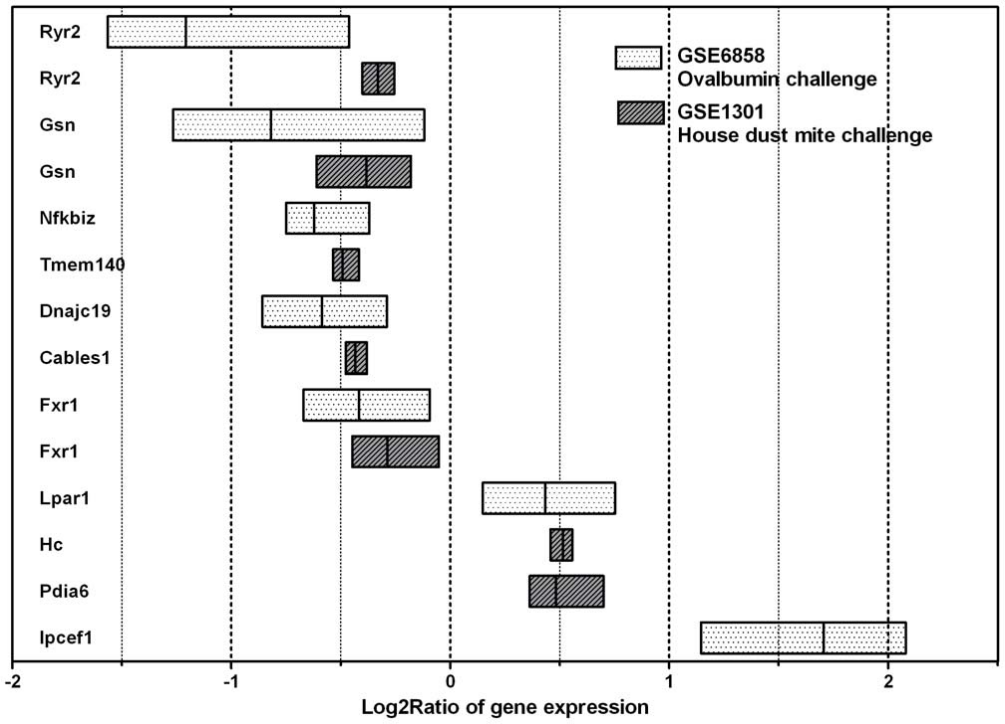
Gene expression profiling of experimental asthma. In two models of experimental asthma induced by Ovalbumin or House dust mite challenge in BALB/c mice there are significant differences in the expression of some genes identified in the GWAS study. Differential gene expression is shown as mean, maximum and minimum of log_2_ ratios of allergen-challenged *vs* control mice.

Biological relevance towards asthma physiopathology was supported by gene expression analysis of experimental asthma mouse models and literature-based functional studies of annotated genes for many of the candidate genetic loci. Many of the genes harboring risk alleles are expressed differentially in asthmatic *vs* control mice in two different experimental asthma models (house dust mite or ovalbumin challenge, **Figure 3**) and have a role in the two key pathways involved in asthma physiopathology (**Table 2**): airway smooth muscle contraction or bronchoconstriction (*RYR2*, *TACR3*, *CHRM2*, *PDE5A*) [30]–[33] and regulation of the pleiotropic mechanisms of inflammation, as modulation of Th2 adaptive immunity and chemotaxis (*C5*, *GSN, IPCEF1*), antigen processing (*CPVL, PDIA6, PTPLB*), cytokine production (*LPAR1*, *FXR1*, *NFKBIZ*) [34]–[42].

**Table 2:** Candidate SNPs associated with allergic asthma onset in a pediatric population.

dbSNP ID	Chr	Minor Allele	Gene Simbol	Description	Risk Allele	Allelic p value	Average cluster Silhouette	Risk genotype	Genotypic p value	ASM contraction	Inflammation	Gene expression
rs25681	9q33.2	T	**C5**	complement component 5	**C**	**0.0011**	**0.577**	**CC**	**0.0045**	**X**	**X**	**X**
rs4679308	3q21.3	C	**CHCHD6**	coiled-coil-helix-coiled-coil-helix domain containing 6	**T**	**0.0249**	**0.536**	**TT**	**0.0564**			
rs334504	7p12.3	C	**TNS3**	tensin 3	**G**	**0.0118**	**0.520**	**GG**	**0.0173**			
rs17456162	2p25.1	G	**PDIA6**	protein disulfide isomerase isozyme A1	**G**	**0.0261**	**0.519**	**AG+GG**	**0.0261**		**X**	**X**
rs10760153	9q33.2	C	**RAB14**	member RAS oncogene family	**T**	**0.0004**	**0.502**	**TT**	**0.0009**			
rs4580655	4q24	G	**TACR3**	tachykinin receptor 3	**G**	**0.0001**	**0.497**	**GG**	**0.0008**	**X**		
rs790259	6q25.2	G	**OPRM1, IPCEF1**	opioid receptor, mu 1-interaction protein for cytohesin exchange factors 1	**A**	**0.0225**	**0.484**	**AA**	**0.0056**		**X**	**X**
rs3250	7q33	C	**TMEM14, C7orf49**	TMEM140/chromosome 7 open reading frame 49	**C**	**0.0033**	**0.477**	**CC+CT**	**0.0004**			**X**
rs1162394	3p25.3	C	**SRGAP3**	SLIT-ROBO Rho GTPase activating protein 3	**C**	**0.0077**	**0.476**	**CC+CG**	**0.0067**			
rs694936	3q12.3	T	**NFKBIZ**	nuclear factor of kappa light polypeptide gene enhancer in B-cells inhibitor, zeta	**A**	**0.0022**	**0.475**	**AA**	**0.0061**		**X**	**X**
rs7792231	7q33	T	**CHRM2**	cholinergic receptor, muscarinic 2	**C**	**0.0002**	**0.459**	**CC**	**0.0001**	**X**		
rs8093359	18q11.2	G	**CABLES1**	Cdk5 and Abl enzyme substrate 1	**G**	**0.0031**	**0.454**	**GG**	**0.01**			**X**
rs12820238	12q21.33	T	**C12orf12, EPYC**	chromosome 12 open reading frame 12/epiphycan	**T**	**0.0019**	**0.450**	**GT+TT**	**0.0018**			
rs2158623	7p15.1	G	**JAZF1**	JAZF zinc finger 1	**T**	**0.0256**	**0.445**	**TT**	**0.032**			
rs2460456	16q24.3	C	**SPG7**	spastic paraplegia 7	**T**	**0.007**	**0.442**	**TT**	**0.0026**			
rs531003	9q31.3	C	**LPAR1**	lysophosphatidic acid receptor 1	**G**	**0.0026**	**0.435**	**GG**	**0.0042**		**X**	**X**
rs506511	7p15.1	T	**CPVL**	carboxypeptidase, vitellogenic-like	**T**	**0.0044**	**0.434**	**TT**	**0.0116**		**X**	
rs6782299	3q26.33	G	**FXR1, DNAJC19**	fragile X mental retardation, autosomal homolog 1/DnaJ (Hsp40) homolog, subfamily C, member 19	**G**	**0.0003**	**0.433**	**GG+GT**	**0.0002**		**X**	**X**
rs10754593	1q43	G	**RYR2**	ryanodine receptor 2	**G**	**0.0003**	**0.431**	**CG+GG**	**0.0016**	**X**		**X**
rs10516997	4p14	C	**APBB2**	amyloid beta (A4) precursor protein-binding, family B, member 2	**G**	**0.0002**	**0.423**	**GG**	**0.0002**			
rs4837827	9q33.2	T	**GSN**	gelsolin	**C**	**0.0011**	**0.412**	**CC+CT**	**0.0056**		**X**	**X**
rs456290	5q33.3	C	**SGCD**	sarcoglycan, delta (35kDa dystrophin-associated glycoprotein)	**C**	**0.0002**	**0.410**	**CC+CT**	**0.0002**			
rs1456114	3q21.1	G	**PTPLB**	protein tyrosine phosphatase-like (proline instead of catalytic arginine), member b	**G**	**0.0032**	**0.403**	**AG+GG**	**0.0023**		**X**	
rs10518329	4q27	G	**PDE5A**	phosphodiesterase 5A, cGMP-specific	**G**	**0.0109**	**0.400**	**AG+GG**	**0.0088**	**X**		

Each cluster of significant SNP is represented here by the one showing the highest silhouette value. Allelic and genotypic *p* values are determined by individual genotyping carried out on the MassArray Sequenom platform. Biological relevance to asthma is shown by literature-based functional classification and gene expression meta-analysis of experimental asthma murine models (ovalbumin or house dust mite challenge). Results are shown by increasing values of allelic *P*. Complete results table, including SNPs not directly associated with Refseq genes (LD r^2^<0.8 on CEPH Hapmap data) are shown in **Table S1–S2 in File S1**.

To identify the candidate genetic loci more likely related to allergic asthma onset, we individually genotyped the 24 top scoring SNPs associated with known genes in an independent validation set. RYR2, CHRM2 and TNS3 polymorphysms were confirmed as associated with allergic asthma onset risk in an independent pediatric population (**Table S3 in File S1**).

## Discussion

We report here the first pooled genome-wide association study of childhood allergic asthma along with the description of an analysis pipeline that efficiently identified candidate genetic loci linked to asthma development. Indeed the pooling approach poses limitations compared to individual genotyping, that range from the inflation of experimental error due to pooling construction, to the inability to adjust for population stratification and the lack of haplotype and subphenotype data. We addressed the first two points by performing multiple technical replicates of each subpool, and by checking the distribution of ancestry-informative SNPs through principal component analysis [24].

GWAS from pooled DNA samples follow generally a two-stage design in which candidates showing putative association are confirmed by individual genotyping [23]. However, while the statistics to identify the most significant candidates are well defined in GWAS from individual samples, there is no widespread agreement on which analysis pipeline should be used to prioritize SNPs for individual genotyping in pooled GWAS. Few studies explored the efficacy of different methods in selecting candidates; among them Pearson *et al.* compared many statistical methods and verified that the silhouette score was the most efficient method to rank SNPs [10]. However, Bossè *et al.* using silhouette statistics on T2DM dataset analyzed by pooled and individual genotyping documented just 30–40% concordance between the two methods [14]. However the authors also claim that a combination of silhouette scores with “absolute difference” testing is superior to silhouette analysis alone for validation studies including fewer than 1000 SNPs. Abraham *et al.* found 68% concordance, with the cluster method based on physical distance on the genome as the single best method [43]. These publications confirm that statistical tests are inadequate to reveal the most pertinent candidate SNPs in a pooled GWAS. While combination with absolute difference can help identifying those markers having the higher allele frequency variations between the two groups (and indeed absolute difference calculated on the 36 significant SNPs reported in our study is high, ranging from 0.086 to 0.24), combination with physical distance on the genome addresses the almost unique feature of SNP association studies, in which statistical association of one marker to a phenotype is expected to be shared with other markers inside a definite LD block. To address these points in the current study we used a modular approach to identify the most significant loci associated with asthma by combining silhouette statistics and physical distance method with cluster-adapted thresholding; in this way we found 97% concordance between pooled and individual genotyping, with 36 out of 37 top-scoring SNP significant at the individual genotyping level.

Moreover the most significant SNP is located inside the coding sequence of C5, that was already identified as an asthma susceptibility gene [29], and the vast majority of the other candidate genes regulate functions that are relevant to bronchial physiopathology, as immune- or inflammation-mediated mechanisms and airway smooth muscle contraction. Lastly, the high correlation with gene expression data in experimental asthma models (42%) further supports the results, since genetic variation is known to influence gene expression [44], and gene expression difference itself was proposed as an efficient method to refine the identification of candidate genes and functions in GWAS experiments [45]. Lastly, three out of 24 candidate SNPs located inside known genes were found significantly associated with asthma in an independent validation set.

In this study we identified candidate genetic variants that distinguish, within a population of allergic children, the subjects with and without asthma; these differences may be due to the differences between nasal and bronchial mucosa physiopathology, that may subtend different genetic pathways between asthma and rhinitis. In fact, even if upper and lower airways may be considered as a unique entity supporting the concept of a “united airways” [46], and are influenced by a common, evolving inflammatory process that may be sustained and amplified by interconnected mechanisms, there are many differences between nose and bronchi: smooth muscle is present from the trachea to the bronchioles explaining bronchoconstriction in asthma, cholinergic nerves are the predominant bronchoconstrictor pathway, α-adrenergic agonists are effective nasal vasoconstrictors in rhinitis whereas β2-adrenergic agonists are effective bronchodilators in asthma and many others.

Differences between children with and without asthma are evident from the analysis of the 269 patients recruited for the study from the clinical and allergometric evaluation; actually asthmatic children have higher total IgE values, higher specific IgE values and higher prevalence of sensitization to house dust mite than patients with RC without asthmatic symptoms. We suggest that these clinical and immunological differences reflect an intrinsic genetic diversity in the immunological mechanism of allergic response.

This is the first study done so far in which asthmatic children have been compared not to the healthy population but to allergic subjects with RC. By considering allergic children as the control group, we excluded any interferences of genes involved in the generic mechanisms of allergy, that mainly regulate the mechanisms of inflammation, alteration of epithelial and mucosal functions and modulation of the immune response to environmental factors, thus identifying only new potential genetic pathway explaining lower airway involvement. The limitation of using the RC population as control group is related to the possible later onset of asthma; however, since the mean age of the patients enrolled in the study is significantly higher than the onset age of asthma (*P*<0.0001), we can consider it a very unlikely event.

The vast majority of the identified candidate SNPs were not previously reported, apart from C5 (*Complement component 5*) whose polimorphisms were already associated with asthma development [29]. The anaphylatoxin C5a is found in high concentrations in the bronchoalveolar lavage fluid of asthmatics and mice with experimentally induced asthma; it can induce smooth muscle contraction, mucus secretion, increased microvascular permeability, leukocyte migration and activation, and degranulation of mast cells [47]. Moreover a very recent publication showed that signalling by complement factor C5a plays a key role in the development and severity of asthma, through the inhibition of IL-17-producing helper T cells and airway hyper-responsiveness [48]. Here we further supported the association on C5 with allergic asthma and furthermore suggested that the entire 9q33.2 region harbouring also Gelsolin and Rab14 is associated with asthma, thus identifying new candidate risk alleles in genes regulating inflammation and immunomodulation.

Indeed almost all of the candidate genes identified up to now belong to functional categories (innate immunity and immunoregulation, Th2 differentiation and effector function, mucosal immunity) implicated both in asthma physiopathology and allergic response in the upper airways and mucosa, very few of the identified genes regulate functions peculiar only of the asthmatic response (bronchial hyperresponsiveness and bronchoconstriction) [1]. Here we suggested for the first time that allergic children that develop or not asthma are genetically different, and that many of the predisposing genes regulate functions (airway smooth muscle contraction) that are uniquely relevant to asthma physiopathology and not to allergy. Indeed the fact that RYR2 and CHRM2 polymorphisms were already confirmed as significant in an independent validation set supports the view that genes regulating bronchoconstrictions should be given more attention than those regulating immune mechanisms that are relevant also to allergic manifestations in the upper airways. However a larger validation study is warranted to better define the most relevant target genes.

In summary, we described a new method of analysis of pooled GWAS data that proved to be efficient in identifying significant candidate SNPs associated to asthma onset within an allergic pediatric population and report a list of candidate susceptibility genes whose genetic variability appears to be associated to an increased risk of asthma development in children already carrying a genetic predisposition to allergic diseases.

## Supporting Information

File S1 Supplementary tables and figures.
(DOC)
