# Supplementary material for: Pooled Genome-Wide Analysis to Identify Novel Risk Loci for Pediatric Allergic Asthma
Source: PLoS One. 2011 Feb 16;6(2):e16912. doi: 10.1371/journal.pone.0016912 (PMC3040188; doi:10.1371/journal.pone.0016912)
Supplement: File S1 — Supplementary tables and figures. (DOC) [file pone.0016912.s001.doc]

## *Supplementary Tables and Figures*

**Pooled genome-wide analysis to identify novel risk loci for pediatric allergic asthma**

Giampaolo Ricci^1^, Annalisa Astolfi^2^, Daniel Remondini^3,4^, Francesca Cipriani^1^, Serena Formica^1,2^, Arianna Dondi^1^, Andrea Pession^1,2^

^1^Pediatric Unit, Department of Gynecologic, Obstetric and Pediatric Sciences, University of Bologna, Bologna, Italy. ^2^Interdepartmental Centre for Cancer Research “G. Prodi”, University of Bologna, Bologna, Italy. ^3^Department of Physics, University of Bologna, Bologna, Italy. ^4^Interdepartmental Centre “L. Galvani”, University of Bologna, Bologna, Italy

**Corresponding author:**

Giampaolo Ricci, Pediatric Unit, Department of Gynecologic, Obstetric and Pediatric Sciences, University of Bologna, S.Orsola-Malpighi Hospital, via Massarenti 9, Bologna, Italy.

Telephone: 00390516363075 – fax 00390516364829

E-mail: [giampaolo.ricci@unibo.it](mailto:giampaolo.ricci@unibo.it)

**Table S1.** SNPs associated with asthma in the allergic population located inside or in LD with known genes. For each SNP the average cluster silhouette value is reported.

**Table S2.** SNPs associated with asthma in the allergic population located in intergenic regions, with no known gene inside the LD block for Hapmap CEU population. For each SNP the average cluster silhouette value is reported.

| **dbSNP ID** | **Chr** | **Cytoband** | **Chr position** | **Minor Allele** | **SNP Type** | **Gene** | **Risk Allele** | **MAF asthma** | **MAF RC** | **Allelic p value** | **Odds ratio** | **CI95** |
| --- | --- | --- | --- | --- | --- | --- | --- | --- | --- | --- | --- | --- |
| rs10754593 | 1 | q43 | 235392971 | G | intron | **RYR2** | G | 0.5 | 0.3 | 0.0087 | 2.38 | 1.24 - 4.59 |
| rs7792231 | 7 | q33 | 136134010 | T | intergenic | **CHRM2** | C | 0.03 | 0.11 | 0.045 | 4.36 | 0.92 - 20.59 |
| rs334504 | 7 | p12.3 | 47545556 | C | intron | **TNS3** | G | 0.23 | 0.38 | 0.0481 | 2.03 | 1.00 - 4.10 |

**Table S3.** SNPs associated with asthma in the allergic population located inside or in LD with known genes significantly associated with asthma onset in the validation set.

A


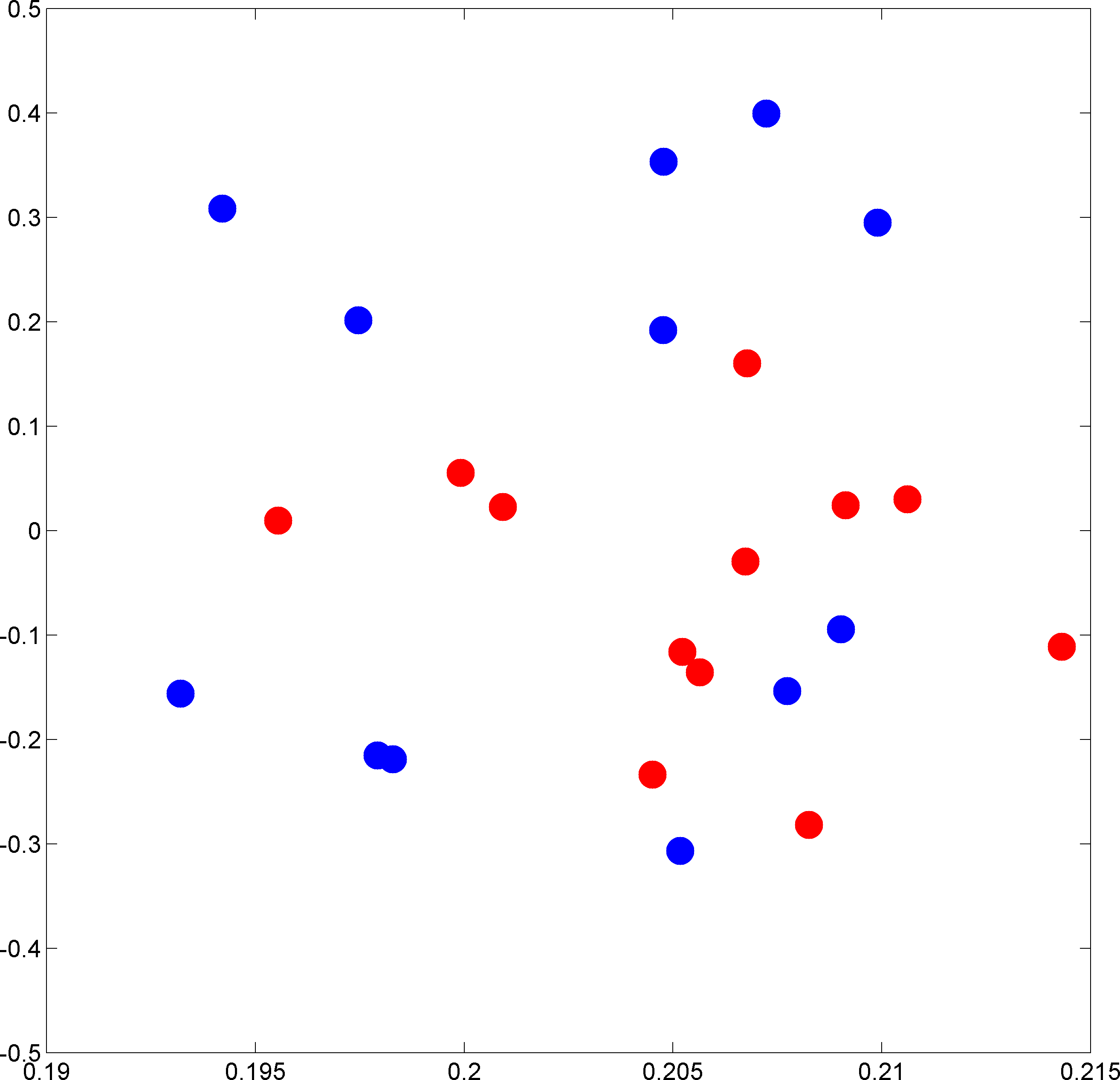


B


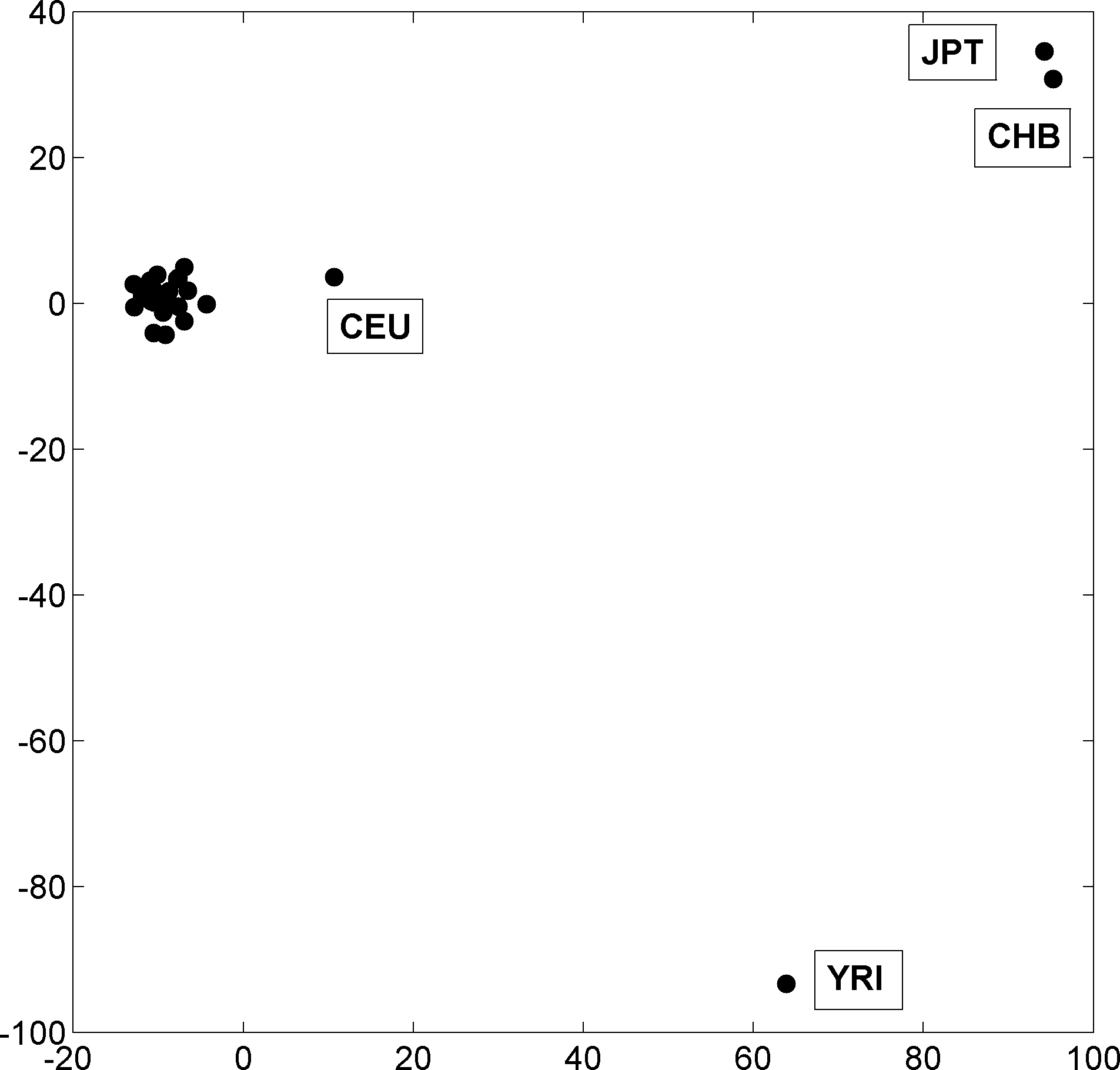


**Pooled data**

**Figure S1.** (a) Principal component analysis of the distribution of RAS values for the Top 500 probes identified as ancestry-related by Drineas *et al*, *PloS One* **5**:e11892 (2010) on the 24 pools analyzed on Mapping 500K Nsp and Sty arrays. Red= asthma; Blue= RC. (b). PCA on the Top 500 probes, compared to allele frequencies measured in CEU, YRI, CHB and JPT individuals (Hapmap data).

A

**
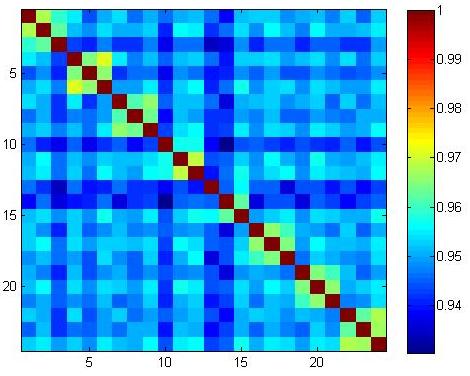
**

B

**
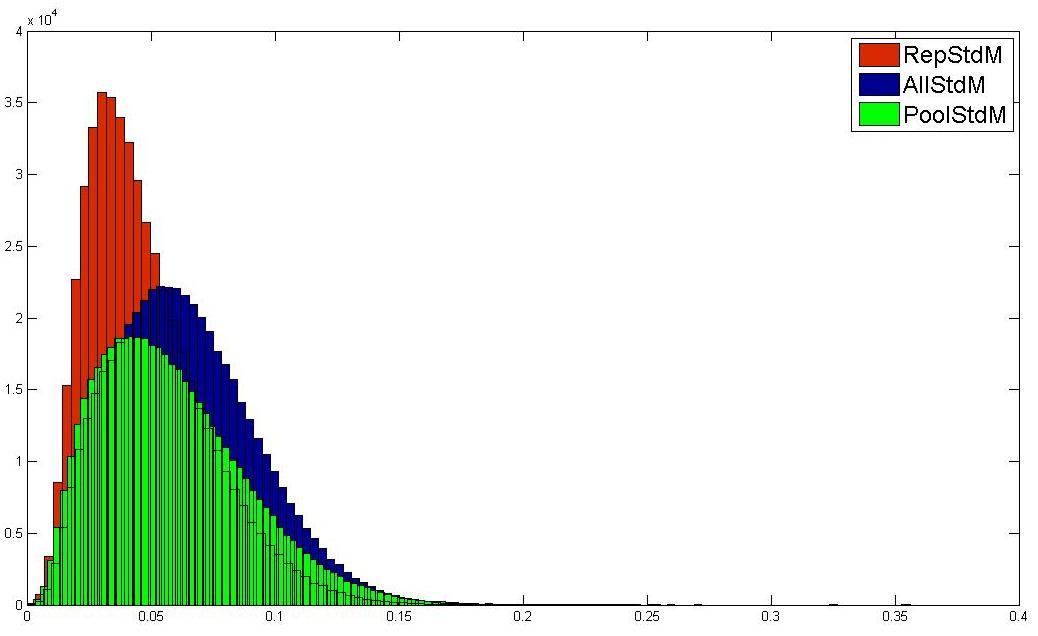
**

**Figure S2.** (a) Pearson correlation for the pooled samples. Technical replicates are close to each other (“yellow” 3x3 squares). (b). Average variance distribution of each probe for the technical replicates (red) for classes (green) and for all arrays (red).


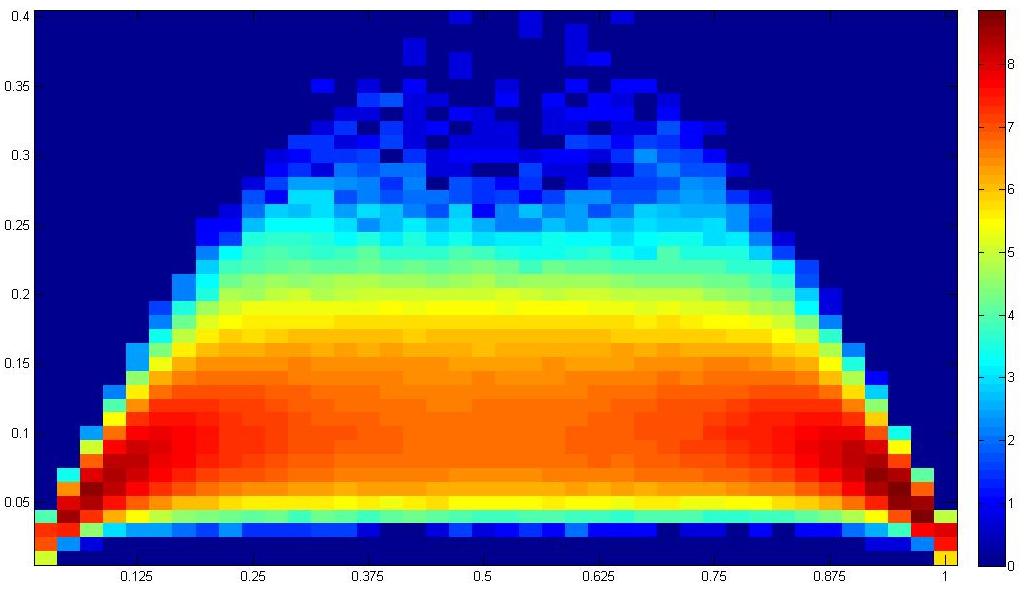


**Figure S3**. Mean RAS (x axis) *vs*. mean AAD (*y* axis) for each probe, 2D histogram.
